# Homozygous delta-beta Thalassemia in a Child: a Rare Cause of Elevated Fetal Hemoglobin

**Published:** 2013-01-22

**Authors:** S Verma, M Bhargava, SK Mittal, R Gupta

**Affiliations:** 1 Senior Resident, Department of Pathology, Chacha Nehru Bal Chikitsalaya, Delhi,India.; 2 Consultant Pathologist, Department of Pathology, Pushpanjali Crosslay Hospital,India.; 3 Director & Senior Consultant, Department of Pediatrics, Pushpanjali Crosslay Hospital,India.; 4 Assistant Professor and Head, Departments of Pathology, Chacha Nehru Bal Chikitsalaya, Delhi,India.

**Keywords:** Delta-Beta Thalassemia, Homozygote, Chromatography, High Pressure Liquid

## Abstract

**Background:**

Delta beta (δβ) thalassemia is an unusual variant of thalassemia with elevated level of fetal hemoglobin (HbF). Homozygous patients of this disorder, unlike β-thalassemia, show mild anemia. Only few cases of δβ-thalassemia have been reported from India in the available indexed English literature.

**Case presentation:**

A four-year old male child was evaluated for recent-onset jaundice. Hematological investigations showed mild anemia with microcytic hypochromic red cells. A comprehensive analysis of hemoglobin by high-performance liquid chromatography (HPLC) showed complete absence of HbA and HbA_2_ with HbF constituting 100% of the hemoglobin. Hemoglobin analysis of both parents showed elevated level of HbF with normal HbA_2_. A final diagnosis of δβ-thalassemia in the child with both parents being carriers was rendered.

**Conclusion:**

Delta beta-thalassemia is an uncommon cause of markedly elevated fetal hemoglobin beyond fetal period. Clinical and haematological parameters should be evaluated to render an accurate diagnosis.

## Introduction

Delta beta (δβ) thalassemia is an infrequent cause of elevated fetal hemoglobin (HbF) beyond infancy. This disorder is characterized by reduction in production of both δ and β-globin chains, usually due to deletions of δ and β structural genes (1). Delta beta (δβ) thalassemia mutations have been reported in various ethnic groups, like Turkish, German, Japanese, Sicilian and Spanish (2). Non-deletional δβ-thalassemia has also been described recently (3). Unlike classical β-thalassemia, the clinical presentation of δβ-thalassemia is mild in both heterozygotes and rare homozygote patients. However, the thalassemic red cell indices in combination with hemoglobin analysis (electrophoresis or HPLC) showing elevated HbF with normal HbA_2_ in heterozygotes and absence of HbA and HbA_2_ in homozygotes help in suggesting the diagnosis (4). Mutation analysis is the confirmatory test for diagnosis of this rare disorder (4). The main differential diagnosis of homozygous δβ-thalassemia includes homozygous hereditary persistence of fetal hemoglobin (HPFH), since both these disorders show 100% HbF(5). The clinical findings of mild anemia with haemolytic features (reticulocytosis, indirect hyperbilirubinemia, and reduced haptoglobin) rules in favour of δβ-thalassemia rather than HPFH(6).Family studies of both parents also show thalassemic red cell indices, with or without anemia and elevation in HbF up to 30% in heterozygotes for δβ-thalassemia(6). Other diagnostic possibilities of 100% HbF include variants of homozygous β-thalassemia and double heterozygosity for δβ-thalassemia and classical β-thalassemia (5,6).

We describe the clinical and haematological features of a young male child with this raredisorder and discuss the relevant literature. 

## Case Presentation

A 4-year male child was referred for further evaluation of acute onset jaundice of 7-8 days duration. There was a history of intermittent diarrhoea since the last four months. However, no other associated urinary complaint was present. There was no history of prior blood transfusion. 

On general examination, the child was thinly built. He was pale and had icterus. Liver was palpable 1cm below right costal margin soft and non-tender, and spleen 3 cm below left costal margin. There was no peripheral lymphadenopathy or ascites. Other systemic examinations were unremarkable. 

**Figure 1:**
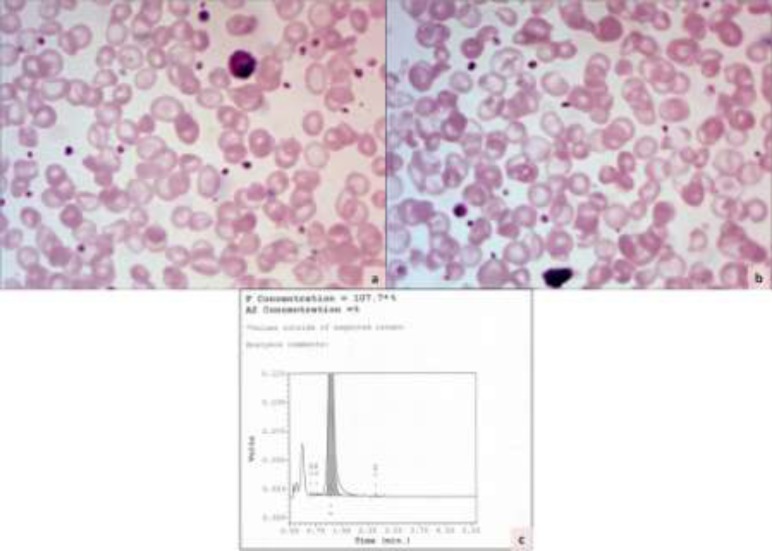
Photomicrographs of peripheral smears of the patient showing microcytic hypochromic red cells with target cells, basophilic stippling and moderate anisopoikilocytosis (a & b, Giemsa x400). HPLC graph demonstrated 100% HbF in the child with absent HbA and HbA_2_ (c

Liver function tests were normal except for an elevated total serum bilirubin (3.5mg /dl) and indirect bilirubin (2.8mg/dl). Hepatitis markers were negative. Serum ceruloplasmin was 28 mg/dl. Hematological investigations revealed mild anemia (hemoglobin 9.8 g/dl) with normal leukocyte and platelet count. Peripheral smear examination showed microcytic hypochromic red cells with target cells and polychromasia (Fig. 1a,b). Few nucleated red cells (4/100 WBC) and basophilic stippling were also noted. Reticulocyte count was high (6%). No microspherocytes or acanthocytes were seen in the blood smear. Further work-up for haemolytic anemia was undertaken. G-6-PD screening test, direct Coombs' test and sickling tests were negative. An HPLC analysis was carried out, which revealed 100% HbF with absence of HbA and HbA_2_ (Fig. 1c). The salient investigations are given in Table I.

**Figure 2:**
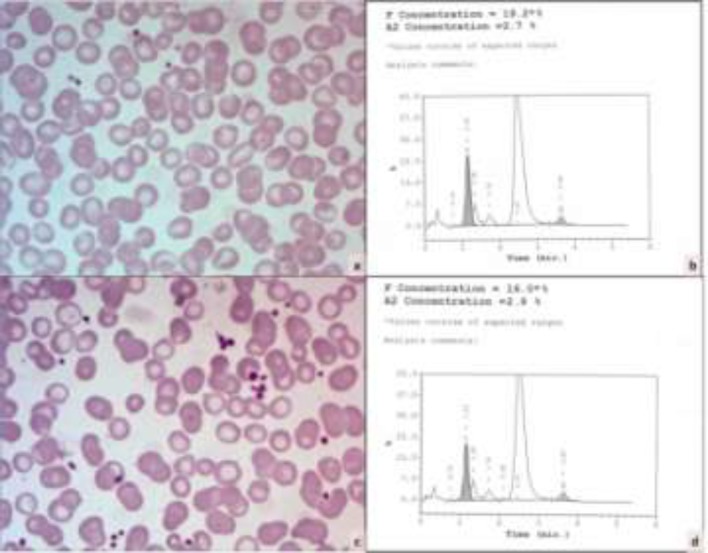
Peripheral blood smear of the father shows few microcytic hypochromic red cells (a, Giemsa x400) while HPLC shows elevated HbF (b). Peripheral blood smear of mother showing microcytic hypochromic red cells and occasional target cells (c, Giemsa x400) and HPLC graph with HbF of 16.0% (d).

Subsequently, HPLC of parents was also performed. Both parents had elevated level of HbF (19.2% and 16.0%) with normal HbA_2_ levels. The smear of both parents also showed hypochromia and target cells (Fig. 2a-d). A history of first-degree consanguinous marriage was elicited. 

In view of the peripheral blood findings of haemolytic anemia with 100% HbF, a diagnosis of homozygous delta-beta thalassemia was rendered and genetic analysis was advised. The genetic analysis could not be performed due to financial constraints. 

**Table I:** Hematological parameters of patient with delta-beta thalassemia

Parameters	Patient	Father	Mother
Hemoglobin (g/dl)	9.8	15.0	12.2
RBC count (million/mm^3^)	4.61	5.84	5.27
MCV (fl) MCHC (pg) RDW-CV (%)	76.4 27.7 17.3	85.0 30.1 15.4	74.2 31.2 17.0
G6PD screening test	NEGATIVE	-	-
HPLC	HbF 100% HbA 0% HbA_2_ 0%	F 19.2% A_2_ 2.7%	F 16.0% A_2_ 2.9%
SGOT (IU/L) SGPT (IU/L)	35 48	-	-
Alkaline phosphatase (IU/L)	144	-	-
S. bilirubin total (mg/dl) Direct bilirubin (mg/dl) Indirect bilirubin (mg/dl)	5.6 0.6 5.0	-	-

MCV: mean corpuscular volume; MCHC: mean corpuscular hemoglobin concentration; RDW-CV: red cell distribution width- coefficient of variation; G6PD: glucose-6-phosphate dehydrogenase; HPLC: high performance liquid chromatography; SGOT: serum glutamate oxaloacetate dehydrogenase; SGPT: serum glutamate pyruvate dehydrogenase; HbF: hemoglobin F (fetal); HbA: adult hemoglobin

## Discussion

Delta-beta thalassemia (δβ-thalassemia), a relatively rare form of thalassemia, is characterized by lack of β and δ-globin chain production (1,4). This reduction in production is usually caused by deletion of δ and β structural genes(7).Non-deletional δβ-thalassemia has also been described, which results from presence in cis of 2 different nucleotide substitutions in promoter of Aγ and β-globin gene(7).Studies of globin chain synthesis have shown that α/non-α chain imbalance in δβ-thalassemia is less pronounced than in β-thalassemia(8). Mutations responsible for δβ-thalassemia have been observed in different ethnic groups, including Turkish, German, Japanese, Black, Sicilian and Spanish type deletion mutations(2). Heterozygotes for δβ-thalassemia are found to have thalassemic red cell indices (high RBC count, low MCV and MCH), normal or reduced HbA_2_ levels and increased amounts of fetal hemoglobin. The HbF is heterogeneously distributed among red blood cells (heterocellular distribution of HbF).^3^ Rare homozygous patients present clinically as either thalassemia intermedia or silent phenotype. Regardless of the clinical presentation, there is thalassemic red cell morphology (microcytic hypochromic anemia with erythrocytosis, high reticulocyte count) along with biochemical evidence of hemolytic anemia (indirect hyperbilirubinemia and reduced serum haptoglobin). This syndrome has also been termed variously as F-thalassemia, β-thalassemia type 2 and normal A_2_ β-thalassemi (9). Earlier authors have described families with homozygous δβ-thalassemic siblings presenting either with mild anemia and hepatosplenomegaly or as clinically normal without anemia or splenomegaly (9,10).The child in the current study was homozygous for δβ-thalassemic and did not have any siblings. However, both of his parents were heterozygous for δβ-thalassemia. Although the exact diagnosis of δβ-thalassemia requires genetic analysis for mutations, hemoglobin electrophoresis or HPLC finding of markedly elevated HbF may be suggestive. Complete absence of HbA with presence of HbF as the single or main hemoglobin component beyond fetal period occurs in four genetic disorders: some variants of homozygous β-thalassemia, some cases of double heterozygosity for δβ-thalassemia and classical β-thalassemia, homozygous patients of hereditary persistence of fetal hemoglobin (HPFH) and homozygous δβ-thalassemia (4,5,11,12). These conditions can be differentiated on the basis of clinical and hematological features. 

Homozygous β-thalassemia usually presents with severe anemia while δβ-thalassemia, even in homozygous patients, shows mild anemia or are in compensated hemolytic process. Homozygotes of HPFH are clinically normal. The normal HbA_2_ levels in both parents exclude the possibility of homozygous β-thalassemia or double heterozygosity for β-thalassemia and δβ-thalassemia in the present case. Hence, the differential diagnosis lies between homozygous δβ-thalassemia and HPFH.

The red cell morphology in the present case (microcytic hypochromic with target cells, polychromasia and reticulocytosis) is suggestive of δβ-thalassemia, since homozygous HPFH shows only a few target cells with normocytic normochromic picture (5). The presence of haemolytic picture in the present case rules out homozygous HPFH. Hemoglobin HPLC of parents, heterozygous for the disorder, showed HbF of 16% and 19%, which are low for heterozygotes of African type HPFH (usually 20-40%) (5). In addition, the red cell indices were of thalassemic type in both parents, again favouring heterozygosity for δβ-thalassemia. 

An extensive PubMed search yielded only few reports of δβ-thalassemia in Indian families, especially from eastern and western India (13,15). Since hemoglobin HPLC is increasingly becoming available at more institutions across the country, this rare disorder should be kept in mind in cases with elevated HbF. 

## Conclusion

This case highlights the importance of considering δβ-thalassemia in presence of elevated HbF and normal or reduced HbA_2_. Careful review of clinical and haematological features assists in differentiation of this rare disorder from HPFH. 
